# Combined compared to dissociated oral and intestinal sucrose stimuli induce different brain hedonic processes

**DOI:** 10.3389/fpsyg.2014.00861

**Published:** 2014-08-07

**Authors:** Caroline Clouard, Marie-Christine Meunier-Salaün, Paul Meurice, Charles-Henri Malbert, David Val-Laillet

**Affiliations:** ^1^INRA, UR1341 Alimentation et Adaptations Digestives, Nerveuses et ComportementalesSaint Gilles, France; ^2^INRA, UMR1348 Physiologie, Environnement et Génétique pour l’Animal et les Systèmes d’ÉlevageSaint Gilles, France; ^3^Agrocampus Ouest, UMR1348 Physiologie, Environnement et Génétique pour l’Animal et les Systèmes d’ÉlevageRennes, France; ^4^INRA, US1395 Ani-Scans, RennesFrance

**Keywords:** cognition, food motivation, reward, brain metabolism, sugar sensing

## Abstract

The characterization of brain networks contributing to the processing of oral and/or intestinal sugar signals in a relevant animal model might help to understand the neural mechanisms related to the control of food intake in humans and suggest potential causes for impaired eating behaviors. This study aimed at comparing the brain responses triggered by oral and/or intestinal sucrose sensing in pigs. Seven animals underwent brain single photon emission computed tomography (^99m^Tc-HMPAO) further to oral stimulation with neutral or sucrose artificial saliva paired with saline or sucrose infusion in the duodenum, the proximal part of the intestine. Oral and/or duodenal sucrose sensing induced differential cerebral blood flow changes in brain regions known to be involved in memory, reward processes and hedonic (i.e., pleasure) evaluation of sensory stimuli, including the dorsal striatum, prefrontal cortex, cingulate cortex, insular cortex, hippocampus, and parahippocampal cortex. Sucrose duodenal infusion only and combined sucrose stimulation induced similar activity patterns in the putamen, ventral anterior cingulate cortex and hippocampus. Some brain deactivations in the prefrontal and insular cortices were only detected in the presence of oral sucrose stimulation. Finally, activation of the right insular cortex was only induced by combined oral and duodenal sucrose stimulation, while specific activity patterns were detected in the hippocampus and parahippocampal cortex with oral sucrose dissociated from caloric load. This study sheds new light on the brain hedonic responses to sugar and has potential implications to unravel the neuropsychological mechanisms underlying food pleasure and motivation.

## INTRODUCTION

Sugars, and especially sucrose, are known to be very attractive in many species. In humans, non-human primates and rats, newborn infants and pups spontaneously respond to sweet taste stimulation with sucrose by specific positive facial expressions (for review, see Berridge, 2000; Steiner et al., 2001). Capaldi and Privitera (2007) also reported that mixing sour or bitter food with the sweet taste of sucrose increased liking for these foods in infant and adult humans, while pigs and rats show spontaneous preferences for sucrose solutions over water (Glaser et al., 2000; Ackroff and Sclafani, 2011). Additionally, adjunction of sucrose as a positive taste reinforcement in a flavored solution induces strong conditioned flavor preferences in rats (Warwick and Weingarten, 1996; Gilbert et al., 2003; Bonacchi et al., 2008), humans (Mobini et al., 2007), and pigs (Clouard et al., 2012a). Interestingly, some studies also suggested that post-oral effects of sucrose, acting independently to oral signals, act as physiological reinforcers and can lead to increased sugar intake. Clear-cut flavor preferences for unsweetened solutions previously paired with intragastric sucrose infusion have indeed been reported in rats (Azzara and Sclafani, 1998; Sclafani and Glendinning, 2005), suggesting that visceral (i.e., gastro-intestinal) sucrose infusion might also be sufficient in itself to induce positive hedonic (i.e., pleasure-related) responses even in the absence of sweet taste. But interactions between the exteroceptive (e.g., taste, odor, etc.) and interoceptive (i.e., visceral) perceptions of food are also important to modulate hedonism, as demonstrated for the alliesthesia phenomenon (Cabanac and Duclaux, 1970; Cabanac, 1971), a phenomenon whereby the pleasantness of an external stimulus depends on the internal state of the organism. Metabolic signals, including gastrointestinal hormones, can modulate central functions that are not only associated with homeostatic regulation of food intake, but also with pleasure, reward and emotion (Shigemura et al., 2004; Uher et al., 2006; Zheng and Berthoud, 2007). As a consequence, exploring sugar-induced pleasure and motivation, and eventually sweet cravings, requires a focus on what is at stake in the brain when an individual is exposed to oral and/or visceral sugar stimulations.

It is now well known that, in addition to the homeostatic regulation of food intake, mainly integrated at the medullohypothalamic level, reward-related brain areas have a major role in the hedonic processing and regulation of food/sugar intake. Functional magnetic resonance imaging (fMRI) studies have shown that processing of oral sucrose signals involved several pleasure-related brain regions, including the insular cortex, striatum (caudate, nucleus accumbens, and putamen), globus pallidus, amygdala, hippocampus, as well as the prefrontal and anterior cingulate cortices in humans (Frank et al., 2008; Smeets et al., 2011; Stice et al., 2013). Recently, Boubaker et al. (2012) investigated the brain responses triggered by duodenal (the duodenum being the proximal part of the small intestine) glucose sensing in pigs using the single photon emission computed tomography (SPECT) and reported metabolic changes in the orbitofrontal and dorsolateral prefrontal cortices, the caudate and putamen, but also in regions that participate in odor processing, including the prepiriform area and the anterior entorhinal cortex. Altogether, these findings emphasize that the processing of both oral and duodenal sugar signals are mediated by both homeostatic and non-homeostatic factors and involve extra-hypothalamic regions that participate in the hedonic regulation of food intake.

Only a few studies compared the brain responses triggered by sweet taste and/or calories during carbohydrate sensing. Chambers et al. (2009), however, reported that oral ingestion of glucose (combining sweet taste and calories), saccharin (sweet taste but no calorie), and maltodextrin (calories but no sweet taste) all induced differential activations in brain structures involved in taste identification and cognitive processes, including the insula and the dorsolateral prefrontal cortex. Only oral glucose and maltodextrin ingestion, however, induced changes in activation in reward-related brain regions, including the orbitofrontal and anterior cingulate cortices and the caudate. Using microdialyse techniques, Ferreira et al. (2012) reported that dopamine levels in dorsal and ventral striatum are associated with the amount of calories ingested in mice, and that striatal dopamine levels fluctuate in proportion to the caloric density of nutrients infused in the gut. Moreover, Ren et al. (2010) also showed in mice that hormonal signaling, rather than glucose utilization, is the main stimulus regulating striatal dopamine release during glucose ingestion, and that intravenous glucose infusions promote the increase of dopamine levels.

All these studies strongly support the assumption that both oral and post-oral signals during carbohydrates ingestion/infusions are processed in pleasure and reward-related brain structures in humans, rodents or pigs. However, as far as we know, no study has yet compared the brain metabolism triggered by oral and/or duodenal sensing of the same sugar (e.g., sucrose) in the aforementioned extra-hypothalamic regions. Both the hedonic oral properties and the post-oral signals arising from sucrose are able to strongly promote food intake and preference. However, little is known about the ability of sucrose intake to stimulate brain reward circuits in the absence of oral signals.

In western countries and, increasingly, in developing countries, modern diets contain a wide array of manufactured and processed foods consisting of mixtures of starch and sugars (for review, Tappy and Le, 2010), with sucrose, fructose, glucose and lactose as the most common dietary sugars. Sucrose remains the leading added sugar consumed in the American diet and the leading source of fructose, although a rapid increase in fructose consumption has been noticed in the last decades (Tappy and Le, 2010). Yet, chronic consumption of high-sugar diets has been proved to induce metabolic, neurophysiological and brain alterations leading to eating disorders and obesity in humans, pigs and rats (Zhao et al., 2005; Lomba et al., 2009; Benton, 2010; Val-Laillet et al., 2010, 2011). Consequently, the characterization of the brain networks that contribute to the processing of oral and/or post-oral sugar signals in animal models, by leading to a better understanding of impaired eating behaviors like the onset of exacerbated sugar preferences in humans, might fulfill the current needs in human nutrition and health research. The pig appears has a very good model for exploring food preferences and brain mechanisms related to nutrition, as stated in previous review papers (Sauleau et al., 2009; Clouard et al., 2012b).

The aims of the present study are (i) to investigate whether the combination (or congruence, i.e., stimulation of two different sensory pathways by similar/consistent stimuli) or dissociation between oral and post-oral sucrose perception influence the brain activity in reward-related brain structures, and (ii) to determine whether duodenal infusion of sucrose in the absence of sweet taste induce specific activity in the brain reward circuit.

## MATERIALS AND METHODS

### ETHICS STATEMENT

The experiments presented in this paper were conducted in accordance with the current ethical standards of the European Union (Directive 2010/63/EU), Agreement No. A35-622 and Authorizations No. 01894 and No. 35-88. The whole experimental procedure presented in this paper has been reviewed and validated by the regional ethics committee in animal experiment of Brittany, France.

### ANIMALS AND HOUSING

A total of seven 30-kg Large White × Landrace female pigs were used in this study. The pigs were housed in individual pens (150 × 60 × 80 cm) and had free access to water. A chain was suspended in each pen to enrich the environment of the animals and fulfill their natural disposition to play. The room was maintained at ∼24°C with a 13:11-h light–dark cycle. The animals were fed daily a pelleted diet composed of 40% pea, 15% corn, 14.46% barley, 13.92% wheat, 13.56% soybean meal, 0.68% calcium carbonate, 0.58% mono-calcic phosphate, 0.3% vegetable oil, 0.3% vitamin complement, 0.24% salt (net energy: 2.15 Mcal/kg of food). The animals received 1 kg of diet per day.

### SURGERY

After a 24-h fasting period, the pigs were preanesthetised with an intramuscular injection of ketamine (15–20 mg/kg, Mérial, Lyon, France). Suppression of pharyngotracheal reflex was obtained by inhalation of isoflurane (3–5% v/v, Baxter SAS, Maurepas, France) immediately before tracheal intubation. A surgical level of anesthesia was maintained by isoflurane (2–3% v/v) delivered by a mechanical ventilator and analgesia was obtained by an intravenous injection of a morphinic agent (Fentanyl 4 ml, 1.4 ml/min, Renaudin, Paris, France). Heart and respiratory rates were continuously monitored throughout surgery using a pulse oximeter (Ohmeda oximeter, GE Healthcare Clinical Systems, Limonest, France) and an infrared capnometer (Amstrong capnometer, Gambo Engström, Bromma, Sweden). A midline laparotomy was performed under aseptic conditions. A catheter was fixed into the proximal duodenum, tunneled under the skin and exteriorized between the shoulders for intraduodenal (ID) infusions of 0.9% NaCl (saline) or 16% sucrose. After surgery, all the animals had 3 weeks of recovery before the start of the brain imaging sessions.

### PRELIMINARY DETERMINATION OF PLASMA GLUCOSE KINETIC

Three 30-kg Large White × Landrace female pigs different from those used in the present study were used to measure plasma levels of glucose further to a 30-min ID infusion of 16% sucrose (300 ml, 197 kcal). The animals were implanted a duodenal catheter following the same procedure as described above (see Surgery). A jugular catheter was also inserted into a collateral vein surrounding the jugular vein in the neck, tunneled under the skin and exteriorized at the nape level. After surgery, all the animals had at least a week of recovery before blood sampling. The day of blood sampling, the pigs were anesthetized after fasting overnight for 16–18 h and intubated following the same procedure as described above (see Surgery). Ears and eyes of the animals were sealed with cotton and surgical tape respectively, in order to minimize auditory and visual stimulations. Light and noise were set to a minimum at least 15 min after the anesthesia and pigs were subjected to a 30-min ID infusion of 16% sucrose (300 ml, 197 kcal). The solution was injected for 30 min with a peristaltic pump connected to the duodenal catheter with an injection rate of 10 ml per min. Blood samples were collected 10 min (-10 min) and immediately before (0 min) the start of the sucrose infusion, every 2 min from 2 to 60 min after the start of the infusion, every 5 min from 60 to 100 min after the start of the infusion and every 10 min from 100 to 120 min after the start of the infusion. Blood samples were collected in tubes containing 5 μl of ethylenediaminetetraacetic (EDTA 0.8 M, Sigma Aldrich, Saint Quentin, France). Blood samples were immediately centrifuged at 4000 g during 10 min at 4°C and the resulting plasma samples were conserved at -20°C until assaying. Plasma glucose was measured in duplicate by an automated spectrophotometric method (Konélab 20i, Thermo Fisher Scientific Inc., Waltham, MA, USA) using the enzymatic assay Glucose RTU^TM^ (BioMérieux^®^ SA, Marcy l’Étoile, France). The intra-assay coefficient of variation was <3%.

### PRELIMINARY EXPOSURE TO ORAL AND/OR DUODENAL SWEET STIMULATIONS

In order to habituate the animals to oral and/or duodenal sucrose stimulations, all the animals were exposed to three different situations twice a week and during the two consecutive weeks before the beginning of the brain imaging sessions. The three experimental situations induced at the moment of the daily meal were: (i) exposure to the standard diet (1 kg, control), (ii) exposure to the standard diet (1 kg) added with 5% sucrose (the added sucrose represented 50 g and 196.9 kcal, which provided oral and visceral exposure to sucrose), and (iii) exposure to the standard diet (1 kg) with concomitant duodenal infusion of a sucrose solution (16%, 10 mL/min during 30 min, 196.9 kcal, i.e., duodenal sucrose stimulation without oral exposure). We chose two different sucrose concentrations for the oral and duodenal stimulations on purpose. A 5% sucrose addition to the feed is sufficient to increase palatability and therefore to induce putative pleasure-related brain activations. Even though 16% sucrose drinking solutions are attractive to pigs (Clouard et al., 2014), a too high sucrose concentration in solid feed might have reverse effects (i.e., aversion) in some animals and would have compromised the feed granulation process and altered the feed texture. For the duodenal stimulation, the aim was to maximize the metabolic effect and homeostatic response (e.g., increased glycemia and insulinemia). A 5% sucrose solution might have been insufficient to induce a significant homeostatic response with clear brain metabolism changes. In contrast, the literature has described clear responses with 16% sucrose infusions.

### BRAIN IMAGING PROCEDURE

During 4 weeks, the pigs underwent four brain-imaging sessions each to investigate the brain metabolism following oral and/or duodenal stimulations with sucrose. The brain imaging modality used to investigate cerebral blood flow (CBF) was the SPECT of technetium-99m (^99m^Tc, CIS Bio International, France) coupled with hexamethyl-propylene-amine-oxime (HMPAO, Ceretec, GE Healthcare, Velizy, France).

#### Animal preparation and oral/duodenal stimulations

After fasting overnight for 16–18 h, the animals were anesthetized in a quiet room and subjected to a tracheal intubation following the same procedure as described above (see Surgery). A venous catheter was inserted into their right ear in order to inject the radiolabel. Light and noise were set to a minimum at least 15 min before the start of the oral and duodenal stimulations. Ears and eyes of the animals were sealed with cotton and surgical tape respectively, in order to minimize auditory and visual stimulations.

**FIGURE 1:**
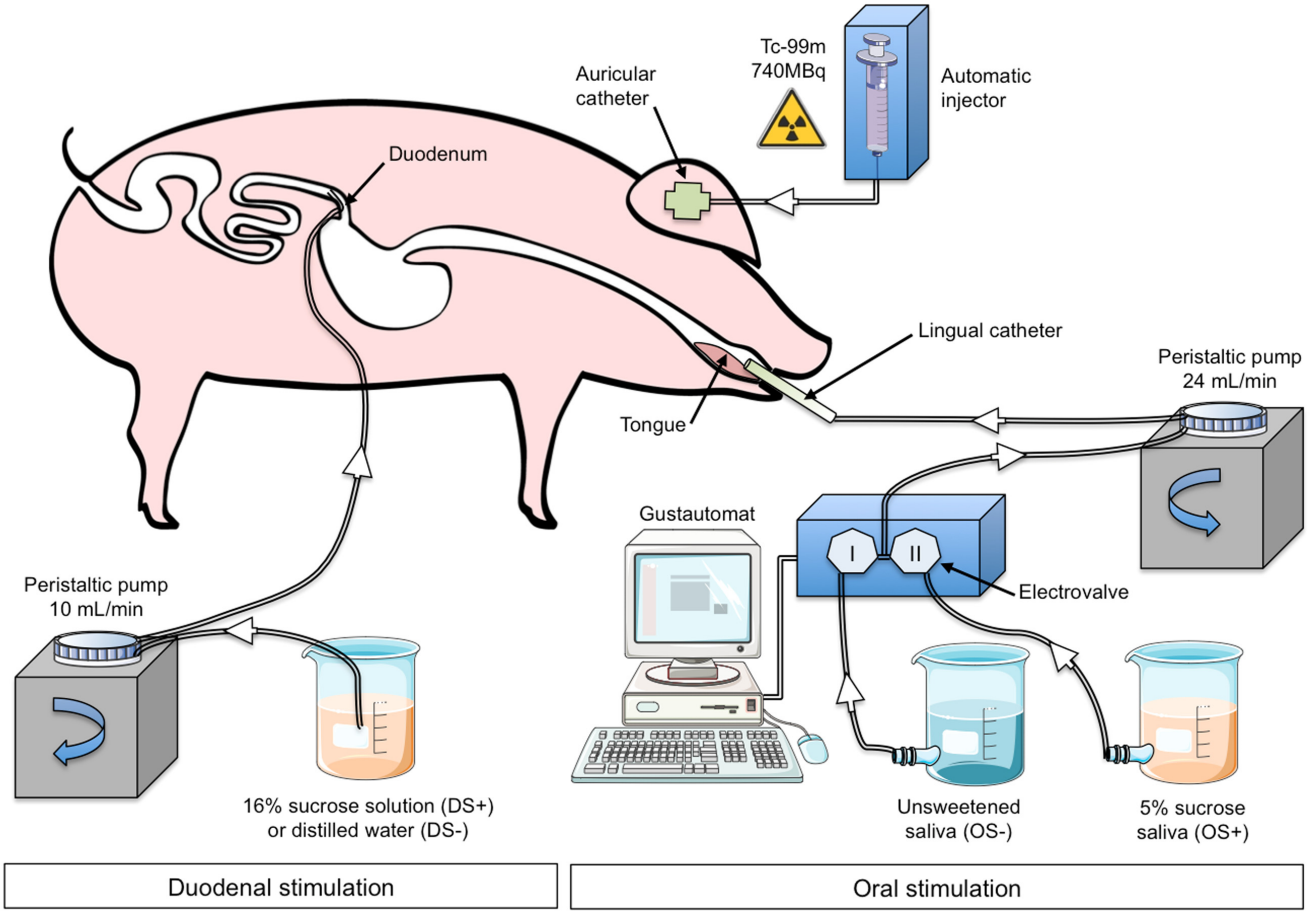
**Experimental device and paradigm designed to perform oral (OS) and duodenal (DS) stimulations for brain imaging in anaesthetized pigs.** The illustrations used to make this figure were obtained from the “Servier Medical Art” website, http://www.servier.fr/servier-medical-art. OS–: oral non-sweet stimulation with artificial saliva; OS+: oral stimulation with 5% sucrose artificial saliva; DS–: duodenal stimulation with saline; DS+: duodenal stimulation with 16% sucrose. Injection of ^99m^Tc-HMPAO was performed 50 min after the start of the DS but was concomitant to the OS.

**FIGURE 2:**
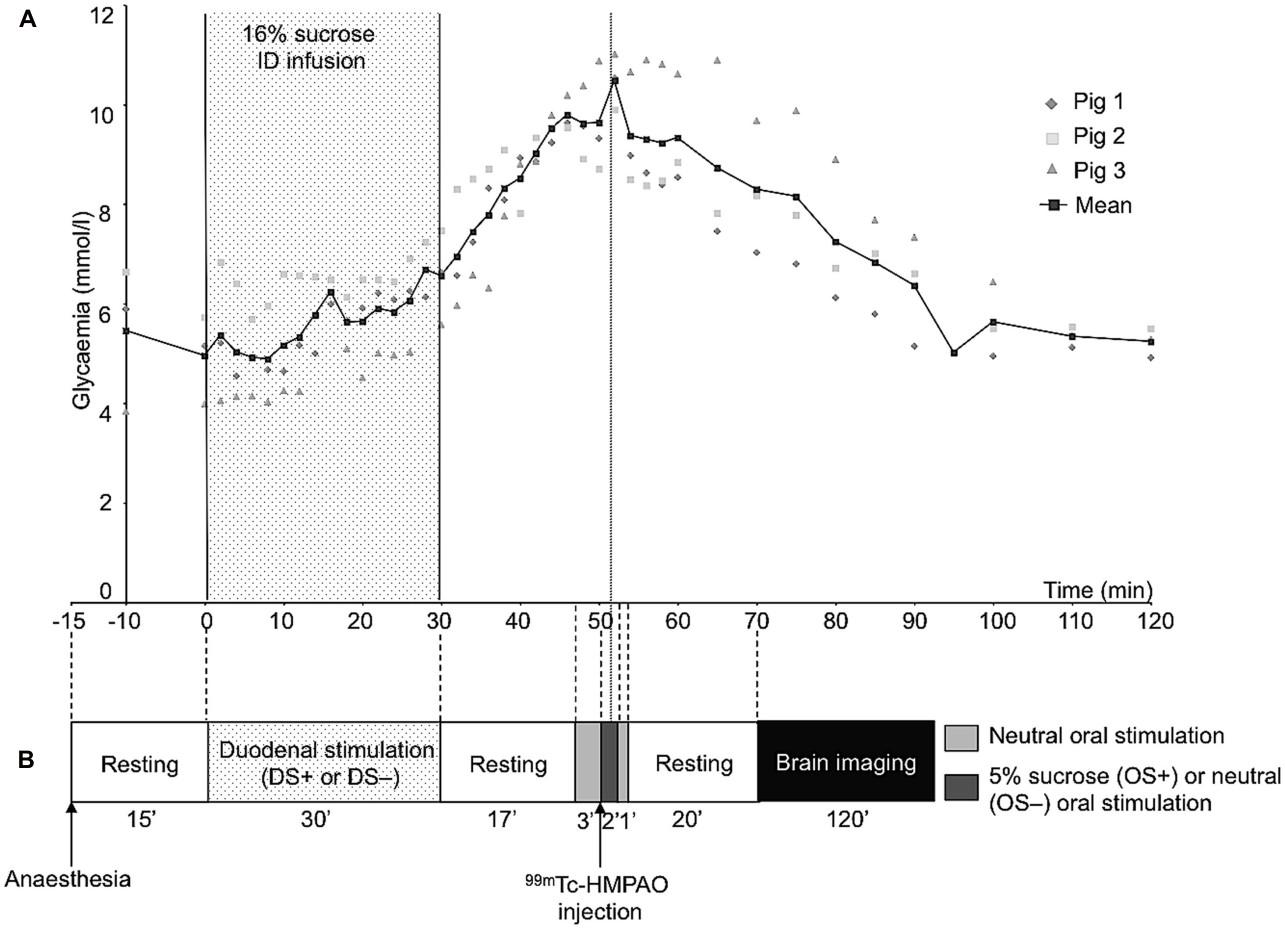
**(A)** Plasma concentrations of glucose before and after a 30-min duodenal infusion of 16% sucrose (10 ml/min, 300 ml corresponding to 197 kcal). Blood samples were collected on three animals different from that used for behavioral tests and cerebral imaging. **(B)** Schematic representation of the experimental paradigm used for the oral and duodenal stimulations before brain imaging. This experiment aimed at exploring the brain metabolism (single photon emission computed tomography of ^99m^Tc-HMPAO) further to oral and/or duodenal sucrose sensing. As the ^99m^Tc has a fast fixation time, the initial tracer uptake reflects cerebral blood flow (CBF) at a fast time window after injection (Kapucu et al., 2009). Consequently, the CBF recorded during imaging, that is 20 min after the radiolabel injection, corresponded to the CBF measured at the time of the radiolabel injection, that is during the oral sensing of artificial saliva added with 5% sucrose (OS+) or unsweetened artificial saliva (OS–, control), and during the peak of maximal glycaemia resulting from the ID infusion of 16% sucrose (DS+) or after ID infusion of saline (DS–, control).

The animal underwent oral (OS) and duodenal (DS) stimulations before brain image acquisition. The experimental devices are illustrated in **Figure 1**. The OS was originally described in Gaultier et al. (2011). It consisted in irrigating the pig’s tongue (24 mL/min) with an unsweetened artificial saliva (OS–) or a sweetened artificial saliva (OS+, 5% sucrose; see Hellekant et al., 1997 for the saliva composition). A tube was positioned on the middle of the tongue and connected to the computer-operated automat developed in our laboratory (Gustautomat, INRA, St Gilles, France) and inspired by the Taste-o-Matic by Hellekant’s group (Danilova et al., 2002). The DS was obtained by ID infusions of solutions. The DS+ corresponded to an ID infusion of 300 ml of 16% sucrose (197 kcal) and the control treatment corresponded to an ID infusion of 300 ml of saline (DS–). The choice to use 16% sucrose for ID infusion was based on the will to obtain a rapid and marked effect of ID sucrose on plasma glycaemia (**Figure 2A**). The solutions were injected with a peristaltic pump connected to the duodenal catheter, and the injection rate was 10 ml per min.

The schematic representation of the experimental paradigm used for the oral/duodenal stimulations before brain imaging is described in **Figure 2B**. The delay between OS and DS was chosen in such a way that the oral sucrose sensing (OS+) and the peak of maximum glycaemia after the ID sucrose infusion (DS+) were synchronized. Briefly, at least 15 min after the anesthesia, the animals were subjected to the DS for 30 min. The OS was performed 47 min after the start of the DS. The OS+ consisted in a 3-min neutral oral stimulation (i.e., unsweetened saliva) to accommodate the mucosa thermoreceptors and mechanoreceptors to the stimulation, preceding the diffusion of sweetened saliva for 2 min. The OS+ was ended by a 1-min neutral stimulation. The OS- consisted in a 6-min neutral stimulation with unsweetened saliva. The order of the four different stimulation treatments (OS+DS+, OS+DS-, OS-DS+, and OS-DS-) was alternated for each pig according to a Latin square procedure to prevent any order effect.

#### Radiolabel administration and image acquisition

As the plasma glucose peak occurs ∼50 min after the start of the DS+ (**Figure 2A**), the radiolabel (^99m^Tc-HMPAO, 740 MBq) was injected 50 min after the start of the DS, that is 3 min after the start of the OS. The radiolabel enters the neurons very quickly after injection (i.e., in less than a minute), proportionally to the neuronal activity, and stays within the neurons, meaning that brain images acquired after the injection of the radiolabel are representative of the brain activity at the moment of the injection.

At least 15 min after the radiolabel injection, the anesthetized animals were transferred and placed in a Head First Prone (ventral decubitus) position on the bed of a gamma-camera (APEX SP-6, Elscint, Tel-Aviv, Israel) fitted with a fan-beam collimator (50-cm focus). SPECT brain image acquisitions were performed at least 20 min after the radiolabel injection, when complete brain–blood equilibrium is reached (Thomsen et al., 2008). Sixty projections with a 120-s exposition were acquired at different projection angles (6°per step). Transaxial images were reconstructed using the filtered back projection method (FBP) applying a Metz filter (power parameter *q* = 3). An acquisition matrix size of 128 × 128 was used and spatial resolution of the final images was 0.76 mm per pixel for *x*- and *y*-axis and 1.47 mm per pixel in *z*-axis.

#### Image processing

The images were processed with statistical parametric mapping (SPM8, Wellcome Trust Centre for Neuroimaging, London, UK) implemented in MATLAB 7.9 R2009B (The Mathworks Inc., Natick, MA, USA). SPM8 software was adapted to the characteristics of the pig brain. Template images based on 16 female pigs of same age and breed, as described in Gaultier et al. (2011), were used as reference images for the processing of the SPECT images acquired in the present study.

In the present study, the actual original DICOM files were converted in NIfTI files with the IMAGEJ software (Wayne Rasband NIH, Bethesda, MA, USA) and the images were manually reoriented. The spatial coordinates were centered compared to a reference point (*x*_0_ *y*_0_ *z*_0_) set at CP (*commissura posterior*) in the CA–CP (*commissura anterior–commissura posterior*) plane according to the stereotaxic reference defined by Saikali et al. (2010). The reoriented images were masked using a large brain mask image to remove the extracerebral matter. The four images of each animal were realigned to the mean using a 6-parameter spatial transformation. The realigned images were spatially normalized using the SPECT template image. Normalization consisted in a 12-parameter affine transformation, followed by a non-linear normalization in 16 iterations. Following normalization, tri-linear interpolation was performed with a final voxel size of 1 mm × 1 mm × 1 mm. Finally, the spatially normalized images were smoothed using a Gaussian smoothing kernel with the full width at half maximum (FWHM) set at 8 mm × mm 8 × 8 mm, and masked using a brain mask image to remove the extracerebral matter. The brain mask image was obtained by thresholding the MRI template, and smoothing this image with a Gaussian kernel filter with FWHM set at 8 mm × 8 mm × 8 mm.

#### Statistical brain imaging analysis

**FIGURE 3:**
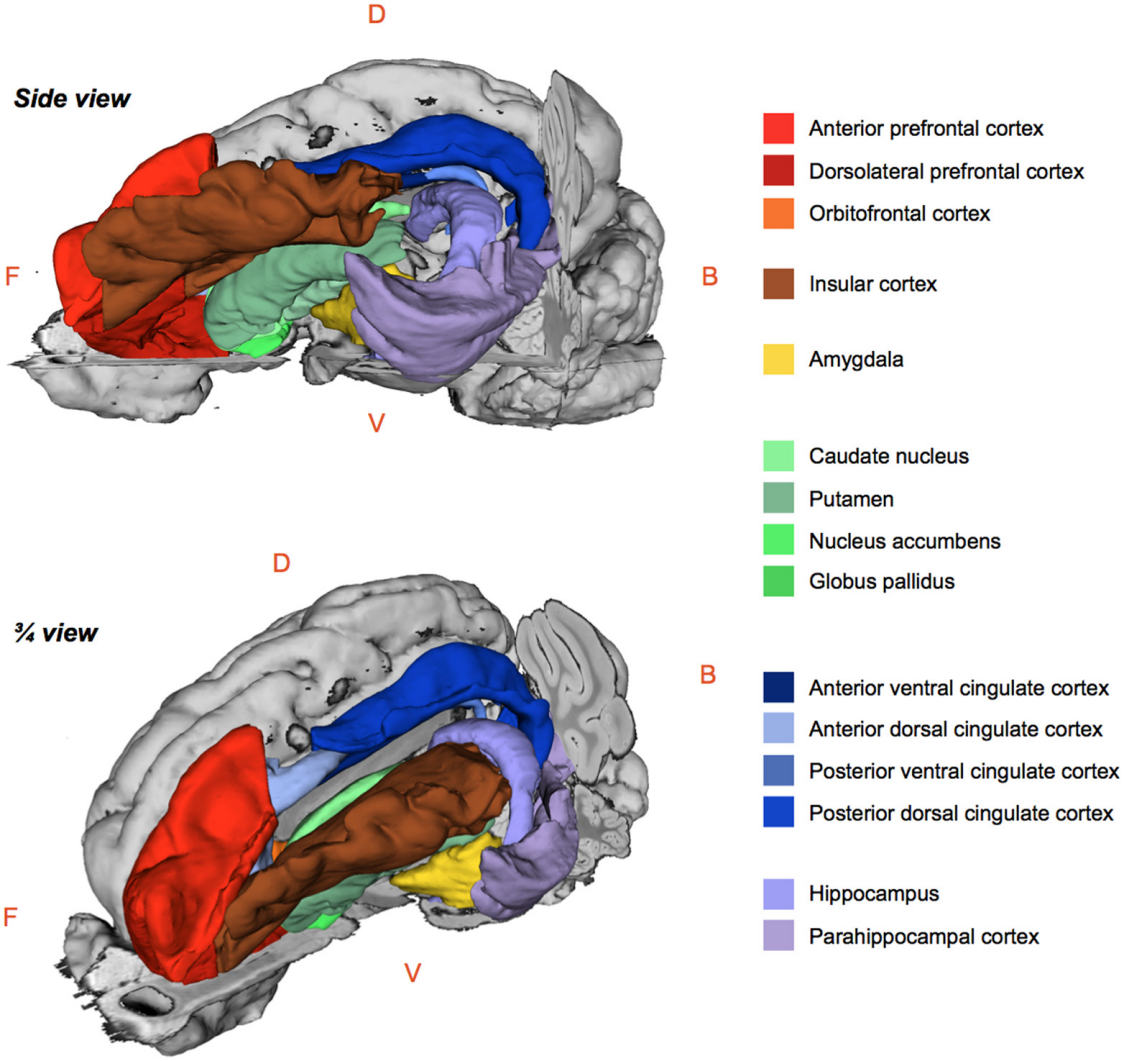
**Schematic 3D-representations of a pig’s brain showing the whole right hemisphere (gray) and a skinned left hemisphere with the regions of interest selected for the brain metabolism small volume correction (SVC) analysis using statistical parametric mapping (SPM).** Images were obtained with 3DSlicer (http://www.slicer.org/) and the 3D-digital pig brain atlas developed in our lab by Saikali et al. (2010). F, Front; B, Back; D, Dorsal; V, Ventral.

The regional ^99m^Tc-HMPAO uptake was standardized to the mean global uptake using proportional scaling. The SPECT images acquired after oral and/or duodenal infusions were analyzed with SPM8 implemented in MATLAB 7.9 R2009B. Paired *t-*tests were performed to compare the effects of oral and/or duodenal sucrose infusions on brain metabolism. A total of three contrasts were analyzed: (1) oral and duodenal sucrose infusions vs. control (OS+DS+ vs. OS–DS–), (2) oral sucrose infusion vs. control (OS+DS– vs. OS–DS–) and, (3) duodenal sucrose infusion vs. control (OS–DS+ vs. OS–DS–). The contrast OS+DS- vs. OS-DS+ was not investigated because it was not possible to dissociate the site effect (oral vs. duodenal) from the concentration effect (respectively 5% and 16% sucrose). A small volume correction (SVC) analysis based on regions of interest (ROIs) selected upon *a priori* hypotheses was performed. This analysis allowed for voxel to voxel comparisons within restricted ROIs corresponding to the cerebral regions in which differential activations were found in previous studies described in the introduction. With this analysis, we managed to identify within specific ROIs the voxels for which the activity was statistically different between treatments. The ROIs included the putamen, the caudate, the globus pallidus, the nucleus accumbens, the amygdala, the insular cortex, the anterior prefrontal cortex, the dorsolateral prefrontal cortex, the orbitofrontal cortex, the cingular cortex (anterior/posterior, dorsal/ventral), the hippocampus and parahippocampal cortex (**Figure 3**). An uncorrected value of *P* = 0.01 was set as the threshold for the clusters’ peak. Clusters comprising a minimum of 25 contiguous voxels were considered significant.

The statistical analysis with SPM8 produced a listing of voxels that corresponded to peaks of maximum intensity for which the CBF differed between treatments in each ROI. Each voxel/peak was associated with a set of coordinates (*x y z*) corresponding to its spatial location in the CA–CP plane with CP set as the origin. The ROIs chosen for the SVC analysis were anatomically identified using a 3D-digitized pig brain atlas developed in our laboratory (Saikali et al., 2010), and representation of the clusters with different metabolism was performed using 3DSlicer (http://www.slicer.org/).

## RESULTS

The results from the preliminary determination of the glucose plasma kinetics are illustrated in **Figure 2A**.

**FIGURE 4:**
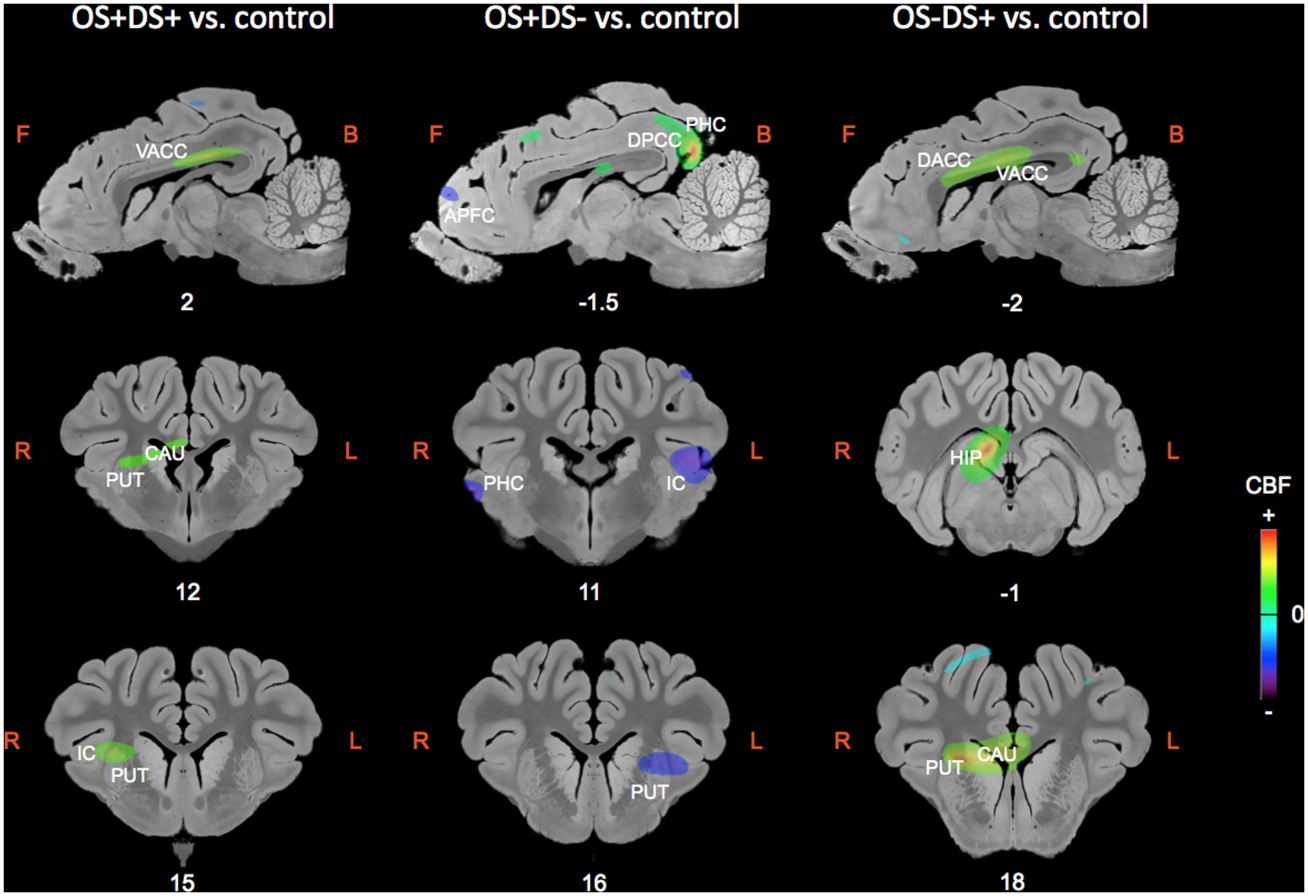
**Sagittal and coronal MRI sections showing clusters of differential cerebral blood flow identified during the small volume correction (SVC) analyses in different regions of interest chosen upon *a priori* hypotheses for the three following contrasts: OS+DS+ (oral and duodenal sucrose stimulation) vs. control, OS+DS- (oral sucrose stimulation) vs. control, and OS-DS+ (duodenal sucrose stimulation) vs. control.** The *x* or *y* coordinates in the CA–CP (*commissura anterior–commissura posterior*) plane are indicated below the images. The threshold for significance was set at *P* < 0.01 (uncorrected). Positive cerebral blood flow (CBF) values indicate a brain metabolism activation and negative CBF values indicate a brain metabolism deactivation compared to control. L, left; R, right; APFC, anterior prefrontal cortex; CAU, caudate nucleus; PUT, putamen; HIP, hippocampus; IC, insular cortex; DACC, dorsal anterior cingulate cortex; VACC, ventral anterior cingulate cortex; PHC, parahippocampal cortex.

The regions of differential CBF values obtained with the SVC analyses on the three contrasts are summarized in **Table 1** and illustrated in **Figure 4**.

**Table 1:** Summary of the brain activations (bold) and deactivations (italic) identified in the regions of interest selected for the small volume correction (SVC) analysis using the SPM (statistical parametric mapping) software (*t* > 3.14; *P* < 0.01 uncorrected) and for the following contrasts: OS+DS+ (oral and duodenal sucrose stimulation), OS+DS- (oral sucrose stimulation only), and OS-DS+ (duodenal sucrose stimulation only) respectively, vs. control.

		OS+DS+ vs. control		OS+DS- vs. control		OS–DS+ vs. control	
Structures	Side	Peak *t*	*x*,*y*,*z* (mm)	Peak *t*	*x*,*y*,*z* (mm)	Peak *t*	*x*,*y*,*z* (mm)
APFC	L	*4.95*	*-6 37 -1*	*4.82*	*-6 36 3*	*4.43*	*-4 33 -4*
APFC	R	*3.62*	*8 42 4*	*3.40*	*4 43 5*		
DLPFC	L	*3.17*	*-10 40 0*	*3.81*	*-2 40 5*		
DLPFC	R	*3.61*	*8 40 3*	*3.84*	*0 41 5*		
				**4.22**	**6 33 14**		
OFC	L						
OFC	R						
							
VACC	L	**3.78**	**-2 4 15**			**5.49**	**0 11 13**
VACC	R	**5.25**	**2 5 14**			**6.74**	**2 9 13**
DACC	L			*3.83*	*-2 39 5*	**4.57**	**0 11 14**
DACC	R			*3.43*	*0 40 4*	**4.69**	**0 11 14**
VPCC	L						
VPCC	R						
DPCC	L			**5.90**	**-2 -12 14**		
DPCC	R			**12.98**	**2 -12 14**		
							
IC	L	*4.28*	*-20 10 11*	*5.08*	*-18 11 10*		
IC	R	**3.60**	**14 17 10**				
							
HIP	L			**3.57**	**-14 4 -6**		
HIP	R	**3.95**	**6 -1 11**			**10.75**	**4 -1 11**
PHC	L			*4.09*	-*12 -9 0*		
				**4.90**	**-16 4 -6**		
PHC	R	*4.92*	*22 8 5*	*5.99*	*22 10 3*	*4.68*	*22 4 1*
				**4.05**	**2 -14 14**		
							
AMY	L						
AMY	R						
							
CAU	L			*3.18*	*-8 18 8*		
CAU	R	**4.47**	**8 7 10**	**3.95**	**6 8 10**	**6.97**	**6 18 8**
PUT	L			*4.33*	*-12 16 7*		
PUT	R	**4.36**	**12 15 9**			**8.97**	**10 19 8**
NAcc	L						
NAcc	R						
GP	L						
GP	R						

*APFC, anterior prefrontal cortex; DLPFC, dorsolateral prefrontal cortex; OFC, orbitofrontal cortex; VACC, ventral anterior cingulate cortex; DACC, dorsal anterior cingulate cortex; VPCC, ventral posterior cingulate cortex; DPCC, dorsal posterior cingulate cortex; IC, insular cortex; HIP, hippocampus; PHC, parahippocampal cortex; AMY, amygdala; CAU, caudate nucleus; PUT, putamen; NAcc, nucleus accumbens; GP, globus pallidus. The peak *t*-value and coordinates in the CA–CP (*commissura anterior–commissura posterior*) reference plane are indicated for each significant cluster, for the left (L) and right (R) hemispheres.*

### ORAL AND DUODENAL SUCROSE INFUSION (OS+DS+) VS. CONTROL (OS-DS-)

Paired oral and duodenal sucrose infusion compared with saline treatment induced peaks of significant activation in the basal nuclei and especially in the right putamen (*t* = 4.36, *P*_unc_ = 0.002, 12 15 9) and right caudate (*t* = 4.47, *P*_unc_ = 0.002, 8 7 10). Peaks of significant activation were also found bilaterally in the ventral anterior cingular cortex (L: *t* = 3.78, *P*_unc_ = 0.005, -2 4 15; R: *t* = 5.25, *P*_unc_ = 0.001, 2 5 14), the right insular cortex (*t* = 3.60, *P*_unc_ = 0.006, 14 17 10), and right hippocampus (*t* = 9.51, *P*_unc_ < 0.001, 6 -1 11). The paired oral and duodenal sucrose infusion compared with saline treatment also induced peaks of significant deactivation bilaterally in the anterior prefrontal cortex (L: *t* = 4.95, *P*_unc_ = 0.001, -6 37 -1; R: *t* = 3.62, *P*_unc_ = 0.006, 8 42 4) and dorsolateral prefrontal cortex (L: *t* = 3.17, *P*_unc_ = 0.01, -10 40 0; R: *t* = 3.61, *P*_unc_ = 0.006, 8 40 3). This contrast was also associated with deactivated clusters in the left insular cortex (*t* = 4.28, *P*_unc_ = 0.003, -20 10 11) and the right parahippocampal cortex (*t* = 4.92, *P*_unc_ = 0.001, 22 8 5).

### ORAL SUCROSE INFUSION (OS+DS-) VS. CONTROL (OS-DS-)

The oral sucrose infusion compared with control treatment induced activated clusters of which the peaks were in the right caudate (*t* = 3.95, *P*_unc_ = 0.004, 6 8 10), right dorsolateral prefrontal cortex (*t* = 4.22, *P*_unc_ = 0.003, 6 33 14), left hippocampus (*t* = 3.57, *P*_unc_ = 0.004, -14 4 -6-), dorsal posterior cingulate cortex (L: *t* = 5.90, *P*_unc_ = 0.001, -2 -12 14; R: *t* = 12.98, *P*_unc_ < 0.001, 2 -12 14) and bilateral parahippocampal cortex (L: *t* = 4.90, *P*_unc_ = 0.001, -16 4 -6; R: *t* = 4.05, *P*_unc_ = 0.003, 2 -14 14). Oral sucrose infusion compared with control also induced peaks of significant deactivation in the left putamen (*t* = 4.33, *P*_unc_ = 0.002, -12 16 7), left caudate (*t* = 3.18, *P*_unc_ = 0.010, -8 18 8), left insular cortex (*t* = 5.08, *P*_unc_ = 0.001, -18 11 10), and bilaterally in the dorsal anterior cingular cortex (L: *t* = 3.83, *P*_unc_ = 0.004, -2 39 5; R: *t* = 3.43, *P*_unc_ = 0.007, 0 40 4), anterior prefrontal cortex (L: *t* = 4.82, *P*_unc_ = 0.001, -6 36 3; R: *t* = 3.40, *P*_unc_ = 0.007, 4 43 5), dorsolateral prefrontal cortex (L: *t* = 3.81, *P*_unc_ = 0.004, -2 40 5; R: *t* = 3.84, *P*_unc_ = 0.004, 0 41 5), and parahippocampal cortex (L: *t* = 4.09, *P*_unc_ = 0.003, -12 -9 0; R: *t* = 5.99, *P*_unc_ < 0.001, 22 10 3).

### DUODENAL SUCROSE INFUSION (OS-DS+) VS. CONTROL (OS-DS-)

Duodenal sucrose infusion compared with saline infusion induced clusters of significant activation with peaks in the right basal nuclei, including the caudate (*t* = 6.97, *P*_unc_ < 0.001, 6 18 8) and the putamen (*t* = 8.97, *P*_unc_ < 0.001, 10 19 8). Peaks of significant activations were also found bilaterally in the ventral (L: *t* = 5.49, *P*_unc_ = 0.001, 0 11 13; R: *t* = 6.74, *P*_unc_ < 0.001, 2 9 13) and dorsal anterior cingular cortex (L: *t* = 4.57, *P*_unc_ = 0.002, 0 11 14; R: *t* = 4.69, *P*_unc_ = 0.002, 0 11 14), as well as in the right hippocampus (*t* = 10.75, *P*_unc_ < 0.001, 4 -1 11). The contrast was also characterized by deactivated clusters of which the peaks were in the left anterior prefrontal cortex (*t* = 4.43, *P*_unc_ = 0.002, -4 33 -4) and right parahippocampal cortex (*t* = 4.68, *P*_unc_ = 0.002, 22 4 1).

## DISCUSSION

The major findings of our study were that oral and/or duodenal sucrose sensing induced CBF changes in brain regions known to be involved in memory, reward processes and hedonic evaluation of sensory stimuli, and that combined (or congruent) oral and duodenal sucrose perception processing differed from that of dissociated oral and duodenal stimulations. Moreover, our results showed that sucrose duodenal stimulation only, which increased blood glucose levels, significantly activated the anterior cingulate cortex, right putamen and hippocampus, similarly to the congruent bimodal sucrose stimulation and contrary to the oral sucrose stimulation only, suggesting that visceral signals can modulate hedonic processes without oral perception. Some brain deactivations in the prefrontal cortex and insular cortex were only detected in the presence of oral sucrose stimulation.

The modulation of the hedonic circuit showed in our study is in accordance with previous work in both fasted and sated humans that revealed metabolism changes in these regions further to sugar ingestion (glucose, Chambers et al., 2009) or during oral taste stimulation paired with caloric load (sucrose, Haase et al., 2009, 2011; Smeets et al., 2011). It is also consistent with previous studies in humans or animal models that highlighted differential brain responses in the aforementioned structures further to separated oral or visceral sugar sensing. In pigs, duodenal glucose infusions in the absence of oral glucose sensing induced differential CBF responses compared to a control stimulation in the prepiriform area, the anterior entorhinal cortex, the orbitofrontal, dorsolateral and anterior prefrontal cortices, the hippocampus and the dorsal striatum (Boubaker et al., 2012). Compared to a control situation, oral sucrose sensing triggered differential activation in the insula and anterior cingulate cortex, the nucleus accumbens and the caudate of sated women (Frank et al., 2008). In fasted humans, ingestion of maltodextrin, a caloric non-sweet compound, triggered activation in the insular, the orbitofrontal and dorsolateral prefrontal cortices and the caudate (Chambers et al., 2009).

The originality and novelty of our work rests upon the comparison of the oral, duodenal and combined stimulations with the same sugar (sucrose) in a controlled experimental model, demonstrating that gustatory and visceral signals can independently or synergistically modulate the hedonic circuit. Some modulations were common to the three contrasts studied (oral/duodenal sucrose vs. control, oral sucrose vs. control, and duodenal sucrose vs. control), such as activation of the right caudate nucleus, or deactivation of the left anterior prefrontal cortex and right parahippocampal cortex. Some other modulations were detected only when duodenal sucrose stimulation was performed, such as the activation of the ventral anterior cingulate cortex, right putamen and right hippocampus. Conversely, some other modulations were detected only when oral sucrose stimulation was performed, such as the deactivation of the dorsolateral prefrontal cortex and left insular cortex. Finally, there was a synergetic effect of combined oral and duodenal stimulation with a specific activation of the right insular cortex.

The dorsal striatum (caudate and putamen) and the prefrontal cortex are both involved in reward processing (Schultz, 2000). The prefrontal cortex, which closely interacts with the striatum, is also known to be involved in the processing of food-related stimulations (Ramnani and Owen, 2004) and to participate in motivation, memory and cognitive functions in humans (Cardinal et al., 2002; Kouneiher et al., 2009). Simons et al. (2005) reported that the anterior prefrontal cortex might participate in memory processes, and more specifically in “the recollection of context detail,” that is in the association between a past event (e.g., food intake) and the contextual information relating to that event (e.g., internal state, subjective feelings, etc.). In the present study, changes in the prefrontal cortex metabolism might be due to the retrieval of contextual information arising from the preliminary exposure to sucrose in awake animals, or simply to sucrose sensing and pleasantness. The anterior prefrontal cortex also shares connections with the dorsolateral prefrontal cortex, which is known to play a major role in the regulation of food intake, notably through its inhibitory inputs to the orexigenic network (Gautier et al., 2000; Del Parigi et al., 2002; Le et al., 2006). Consequently, the perception of the sweet taste of sucrose in the mouth might stimulate appetite through the deactivation of the dorsolateral prefrontal cortex. Further behavioral studies in the pig model or human are necessary to confirm this hypothesis, but it is already well known that sweet taste increases food palatability and appetence in pigs and humans (Kampov-Polevoy et al., 2006; Clouard et al., 2012b).

The activation of the putamen was only visible when duodenal sucrose infusion was performed (with or without concomitant oral sucrose), suggesting that sugar ingestion rather than sweet taste in itself is probably responsible for the significant activation of the putamen further intake of high-sugar beverages in humans (Stice et al., 2013). The fact that oral perception of sugar, without calorie ingestion or duodenal sugar sensing, activates the dorsal striatum differently from the other treatments raises the question of the congruence between oral and visceral sweet perceptions. It is well known that non-caloric sweeteners activate the brain reward circuit differently from caloric sweeteners like sucrose, and that chronic consumption of sweeteners can alter the brain processing of sweet taste (Green and Murphy, 2012). Frank et al. (2008) demonstrated that sucrose elicits a stronger brain response in the anterior insula and striatum compared to sucralose, a non-caloric sweetener. Moreover, only sucrose engaged dopaminergic midbrain areas in relation to the behavioral pleasantness response, and the taste pleasantness predicted left insula response (Frank et al., 2008). This is consistent with our own data that showed (i) a consistent activation of the dorsal striatum only when sucrose sensing was accompanied by a caloric intake (and elevated plasmatic glycemia), and (ii) metabolism changes in the left insular cortex only when sucrose was perceived orally. In a nutshell, these results suggest that even if sweet taste only might induce pleasantness, its combination with a congruent visceral signal (caloric load and elevated glucose plasma levels) is necessary to trigger the activation of anterior cingulate cortex and putamen. This could have important implications on how effective non-caloric sweeteners are in their ability to substitute sugar intake. Their impact on appetite, food intake and motivation should be explored more precisely, both in humans and animals models.

The absence of brain metabolism differences between treatments in the nucleus accumbens is quite surprising though. The nucleus accumbens is a well-known hotspot for food liking and wanting (Berridge, 2009), and functional anomalies of this nucleus, that are characteristic of drug-addicted patients, were described in obese humans (Wang et al., 2001; Volkow et al., 2008) as well as in minipigs fed a high-fat and high-sugar diet (Val-Laillet et al., 2011). Avena (2007) and Avena et al. (2008) consistently and elegantly demonstrated the neurochemical addictive-like responses to sugar in rodents, and especially the patterns of dopamine release in the nucleus accumbens induced by intermittent and excessive sugar intake. Interestingly, Avena et al. (2006) also demonstrated *via* a sucrose sham feeding procedure that dopamine is repeatedly released in the nucleus accumbens in response to sweet taste, as previously showed by Mark et al. (1991) with saccharin, and that acetylcholine is released while drinking sugar in real-feeding rats, but not in sham-feeding rats. The fact that dopamine/opioid stimulation of nucleus accumbens can amplify the reactivity of mesocorticolimbic circuits and consequently magnify incentive salience of cues associated with sucrose reward might explain the brain functional anomalies and increased “wanting” observed further chronic sugar intake. Of course, the absence of CBF differences in the nucleus accumbens in our animals does not preclude the possibility of differences in term of neurotransmitters metabolism and activity, that could be investigated *via* PET in pigs (Alstrup and Smith, 2012). All these results, including ours, suggest that the ventral and dorsal striatum have specific roles in integrating taste and metabolic signals related to sugar consumption, and that further functional and molecular brain imaging studies in rodent and pig models should help disentangling their respective roles in the onset of addictive-like behavior and deregulation of food intake control further to chronic overconsumption of sugars.

The hippocampus and parahippocampal cortex are known to be involved in the integration of subjective internal states with relevant sensory cues (LaBar et al., 2001). It is interesting to notice that, if no difference of metabolism was observed in these regions between the two contrasts including duodenal sucrose stimulation, the perception of oral sucrose only induced many specific changes of metabolism. The same applies to the dorsal posterior cingulate cortex. These findings support the idea that the hippocampus and parahippocampal cortex might contribute to the integration of congruent/incongruent nutrient sensing, as suggested by Haase et al. (2009), i.e., in our study, a sweet taste in the mouth but with no calorie and no sucrose receptors activation in the intestine. In rodents, the perirhinal cortex, which is part of the parahippocampal cortex, sends sensory information to the hippocampus (Furtak et al., 2007; Kerr et al., 2007). The hippocampus supports memory and cognitive functions, while participating in emotional processes (for review, see Fanselow and Dong, 2010). Consequently, the CBF changes observed in the hippocampus and parahippocampal cortex showed that brain regions involved in memory and emotion mediated the processing of oral and/or duodenal sucrose sensing differently. The fact that the metabolism changes in the hippocampus and parahippocampal cortex were similar between combined oral/duodenal sucrose and duodenal sucrose, but different from the activation/deactivation pattern induced by oral sucrose only, comforts the idea that these two structures are probably important to recognize the congruence of a sweet oral stimulation according to the internal state, or that recall processes related to sucrose sensing might be independent of duodenal signals. In a further study, it would be interesting to homogenize the sucrose concentrations used for oral and intestinal stimulations because in the present work, the sucrose solution infused in the duodenum was more concentrated than that infused in the mouth. This methodological limitation prevented us to compare oral sucrose only vs. duodenal sucrose only, and we cannot exclude that some brain metabolism changes observed with the combined stimulation are perhaps due to the perception of incongruence in terms of solutions strength.

The large overlapping of brain metabolism between the two conditions involving a visceral stimulation are probably partly related to the internal state of the subjects, since brain imaging was performed when glucose plasma levels (that mediate early satiation) were at a maximum. Numerous studies reported that brain activation is modified by the internal state of the subjects (hunger or satiety) at the time of imaging. Using PET, Tataranni et al. (1999) found an increased CBF in regions involved in the homeostatic regulation of food intake (e.g., hypothalamus), in taste recognition and hedonic evaluation (e.g., insula and orbitofrontal cortex), in emotion and memory processing (e.g., parahippocampal and anterior cingulate cortices, caudate, putamen and hippocampus) in a hunger state compared to a situation where the subjects had ingested a caloric meal before imaging (satiety state). Using fMRI, Haase et al. (2011) reported that, in hungry and sated humans, sucrose ingestion induced changes in activation in the cingulate, orbitofrontal and parahippocampal cortices, the amygdala, the insula and the hippocampus. Haase et al. (2009, 2011) also reported that there were significantly greater responses in the hunger than in the satiety condition during sucrose stimulation in taste regions (the insula and the orbitofrontal cortex), in regions that participate in emotion processing (the amygdala, the caudate and the cingulate cortex) and memory (the hippocampus and the parahippocampal cortex). Altogether, these findings suggest that internal state plays a major role on brain activation during food-related stimulations.

## CONCLUSION AND PERSPECTIVES

In conclusion, we demonstrated that oral, duodenal and the bimodal perception of sucrose induced different patterns of brain metabolism in structures involved in memory, reward processes and hedonic evaluation of sensory stimuli. Using controlled conditions in a pertinent animal model for human nutrition and nutrient sensing, we managed to demonstrate that some brain metabolism changes are specific to oral sensing or duodenal sensing, respectively, and that bimodal sucrose stimulation can even have a synergetic effect in some brain areas such as the insular cortex. We identified brain areas that are probably involved in the congruence between the sweet perception and internal state. All these results have important implications for discussions related to caloric vs. non-caloric sweeteners consumption and impact of sugars on the brain hedonic circuit and motivational processes.

To disentangle the respective roles of oral and post-oral signals in reward-related responses and their anomalies, further work would benefit from combining different brain imaging modalities to investigate brain activity (assessed *via* CBF or glucose consumption) in conjunction with molecular explorations (e.g., brain dopamine and opioids). Also, because sugar consumption is frequently associated with fat in western diets, it is important to examine the relative role of sugar and fat-food contents in the activation of brain reward regions, as well as their possible interaction/synergy in promoting brain anomalies and at-risk eating behaviors. From a recent study performed in humans (Stice et al., 2013), it appears that sugar more effectively recruits reward and gustatory brain regions than fat, which justifies conducting controlled studies with brain imaging in animal models to confirm these findings and explore the underlying mechanisms.

## AUTHOR CONTRIBUTIONS

Conceived and designed the experiments: David Val-Laillet, Marie-Christine Meunier-Salaün and Caroline Clouard. Performed the experiments: Caroline Clouard. Analyzed the data: Caroline Clouard, Paul Meurice and David Val-Laillet. Contributed reagents/materials/analysis tools: Charles-Henri Malbert and Paul Meurice. Wrote the paper: Caroline Clouard and David Val-Laillet. Revised the paper before submission: David Val-Laillet, Marie-Christine Meunier-Salaün, Charles-Henri Malbert and Paul Meurice.

## Conflict of Interest Statement

The authors declare that the research was conducted in the absence of any commercial or financial relationships that could be construed as a potential conflict of interest.
